# Immediate Effects of Functional Electrical Stimulation-Assisted Cycling on the Paretic Muscles of Patients With Hemiparesis After Stroke: Evidence From Electrical Impedance Myography

**DOI:** 10.3389/fnagi.2022.880221

**Published:** 2022-05-16

**Authors:** Le Li, Chengpeng Hu, Kenry W. C. Leung, Raymond K. Y. Tong

**Affiliations:** ^1^Institute of Medical Research, Northwestern Polytechnical University, Xi’an, China; ^2^Department of Biomedical Engineering, The Chinese University of Hong Kong, Hong Kong, Hong Kong SAR, China

**Keywords:** cycling, electrical impedance myography (EIM), muscle, stroke, functional electrical simulation (FES)

## Abstract

**Background:**

Electrical impedance myography (EIM) has been applied to assess muscle health conditions in neuromuscular disorders. This study aimed to detect immediate muscle electrical impedance property alterations in lower extremity of chronic stroke survivors immediately after functional electrical stimulation (FES)-assisted cycling training.

**Methods:**

Fourteen chronic stroke survivors were recruited for the current study. EIM measurements were conducted before and immediately after 40-min FES-assisted cycling training for each subject. Four interested muscle groups [rectus femoris (RF), biceps femoris (BF), tibialis anterior (TA), and the medial head of gastrocnemius (MG)] were selected. Correlation analysis was performed to reveal a significant correlation between changes in EIM parameters and clinical scales [Fugl–Meyer Assessment of the lower extremity (FMA-LE); 6-min walking test (6MWT)].

**Results:**

Immediately after training, reactance (*X*) and phase angle (θ) values significantly increased on the TA and MG muscles. Significant correlation was observed between *X* value and FMA-LE scores (*r* = 0.649, *p* = 0.012) at MG as well as *X* and FMA scores of the ankle joint (*r* = 0.612, *p* = 0.02). Resistance (*R*) and θ were significantly correlated with 6MWT score (R-6MWT: *r* = 0.651, *p* = 0.012; θ-6MWT: *r* = 0.621, *p* = 0.018).

**Conclusion:**

This brief report demonstrated that EIM can reveal the intrinsic property alteration in the paretic muscle of chronic stroke survivors immediately after FES-assisted cycling training. These alterations might be related to muscle hypertrophy (i.e., increases in muscle fiber size). This brief report might aid the understanding of the mechanism of electrical stimulation-assisted exercise in improving muscle function of stroke survivors.

## Introduction

Stroke is reported to be the second-largest cause of death worldwide (GBD 2016 Stroke Collaborators, 2019), in addition to causing substantial sensorimotor dysfunction and restricting the performance of daily life activities in survivors (Scherbakov et al., 2013). The locomotion-related abilities needed for daily activities are affected by neurological deficits, poor motor control, and uneven muscle stiffness post-stroke (Chen et al., 2003; Peri et al., 2016). Muscle weakness and uncoordinated contraction are among the main targets of the rehabilitation process for the lower extremities in stroke survivors.

Functional electrical stimulation (FES) and cycling training are common rehabilitation interventions used to improve locomotion function in chronic stroke survivors. These interventions help reduce muscle spasticity and improve muscle function (Raasch and Zajac, 1999; Howlett et al., 2015). FES is a common rehabilitation intervention used to improve motor function in stroke survivors. It involves applying short bursts of electrical pulses on the intact peripheral motor nerves to elicit action potentials that propagate along the axons toward the target muscle. The electrical pulses can facilitate the voluntary contraction of paretic muscles to generate functional movement. Moon revealed that muscle tone and stiffness of the ankle dorsiflexor muscle were reduced after an FES intervention (Moon et al., 2017). A study that combined FES with locomotion-like movement revealed a positive effect on gait speed and muscle spasticity immediately after 20 min of training (Yamaguchi et al., 2012). However, the alteration of muscle properties that underpins functional improvement remains underexplored. For example, it was reported that FES had a critical limitation in that it rapidly induces muscle fatigue due to its highly synchronized activation of muscle fibers (Bickel et al., 2011). Therefore, a quantitative evaluation of the muscle changes induced by stimulation and exercise is warranted.

Electrical impedance myography (EIM) is a non-invasive tool used for the assessment of muscle conditions in neuromuscular disorders. During EIM measurements, a series of weak multifrequency currents is passed through the muscle of interest *via* electrodes. The resultant voltage within the local area is then measured. Reactance (*X*), resistance (*R*), and the phase angle (θ) are calculated to determine the intrinsic muscle properties, including the amount of extra- and intracellular fluids in the muscle, the location of tissue interfaces, and cell membrane integrity (Shiffman et al., 1999; Mulasi et al., 2015). In our previous study, EIM was applied to determine the effects of muscle fatigue during and immediately after sustained muscle contractions. It was found that the change in resistance was related to the accumulation of metabolites in the muscle tissue after contraction (Li L. et al., 2016). A study conducted in mice utilized EIM as a tool to assess muscle changes after electrical stimulation. The results revealed significant changes in the *X*, *R*, and θ values when compared to the baseline assessment (Li J. et al., 2016). However, there is limited knowledge on the immediate effects of electrical stimulation training on spastic muscles in stroke survivors. Therefore, determining the electrical property changes in muscle induced by FES using EIM is a relevant area of study. The findings might be helpful for investigating internal morphologic alterations in muscle (Shiffman et al., 2003), changes in the intramuscular environment (body cell mass and intracellular water) (Huang et al., 2014), and physiological metabolite accumulation (Li L. et al., 2016) due to stimulation or muscle contraction.

This study aims to use EIM to detect immediate changes in muscle impedance properties after FES-assisted cycling training in people with chronic stroke. Our hypothesis is that EIM can reveal transitory changes in exercised muscle after one training session. The alterations present immediately after an intervention might provide insights for the design of clinical interventions involving FES for stroke survivors (including time window and intensity) and may help to reveal the mechanism behind FES-assisted cycling training in stroke.

## Methods

### Study Design

This was a self-controlled pilot study that comprised part of an ongoing randomized clinical trial. The inherent muscle properties of the rectus femoris (RF), biceps femoris (BF), tibialis anterior (TA), and medial head of gastrocnemius (MG) were recorded by EIM. The study was reviewed and approved by the Joint CUHK-NTEC Clinical Research Ethics Committee (Ref. no: 2016.093-T). The study was conducted in accordance with the Declaration of Helsinki. All recruited subjects provided written consent before the experimental procedures began.

### Participated Subjects

A convenient sample of fourteen persons with stroke (9 women and 5 men, mean age of 57.19 years old) was recruited in this study. The time since stroke ranged from 3 to 29 years (Table 1). None of the participated subjects had a known history of any other neuromuscular diseases or neurological disorders. The inclusion criteria were as follows: (1) first-ever stroke with unilateral hemiparesis; (2) hemiparesis over 6 months after a stroke; (3) available range of motion at the bilateral ankle joints from 10° dorsiflexion to 30° plantar flexion, at the bilateral knee joints from 0 to 150° flexion, and at the hip joints from 0 to 100° flexion; (4) sufficient cognitive function to understand and perform corresponding tasks; and (5) no history of traumatic injury or surgery of the lower limbs. The exclusion criteria were as follows: (1) high spasticity of the muscles of the ankle, knee, and hip joints; (2) inability to exercise on a cycle ergometer; and (3) antispastic medication use within half a year prior to the study.

**TABLE 1:** Clinical information of subjects.

		MAS								
		
ID	Gender	Knee (F/E)	Ankle (PF/DF)	Stroke type	Paretic side	Age (y)	Duration (y)	FMA-LE	FMAac	6MWT
1	Female	1/0	2/1	Ischemic	Left	60–65	8	25	17	280
2	Female	1/1	1/1	Ischemic	Right	55–60	3	30	11	260
3	Female	1/1	2/1	Hemorrhagic	Left	65–70	10	25	13	224
4	Male	2/2	1/2	Ischemic	Left	65–70	11	19	11	308
5	Female	2/1	2/2	Ischemic	Left	55–60	7	24	8	304
6	Female	2/1	2/2	Hemorrhagic	Right	65–70	8	20	10	185.5
7	Male	2/1	2/1	Ischemic	Right	35–40	5	25	10	295.3
8	Male	2/2	3/3	Hemorrhagic	Right	55–60	7	16	10	244.5
9	Female	1/1	1/1	Ischemic	Left	70–75	5	21	5	172.9
10	Male	1/1	2/2	Ischemic	Left	60–65	3	25	9	170
11	Male	1/1	1/1	Ischemic	Left	55–60	3	28	9	366
12	Male	2/1	1/1	Ischemic	Right	60–65	5	21	14	315
13	Female	0/1	0/1	Hemorrhagic	Right	35–40	3	17	9	188.8
14	Female	1/1	1/0	Ischemic	Right	60–65	4	20	6	264
Mean						58.9	5.9	22.6	10.1	255.6

*FMA-LE, fugl–meyer assessment of the lower extremity; FMAac, FMA score of ankle joint and coordination; MAS, modified Ashworth scale; F, flexion; E, extension; PF, plantar flexion; DF, dorsiflexion.*

### Procedures

This study utilized EIM to assess inherent property alterations in the RF, TA, BF, and MG immediately after FES-assisted cycling training. The EIM assessment was performed on the bilateral RF, TA, BF, and MG before and after a 40-min FES-assisted cycling training session.

### Clinical Assessments

Clinical assessments, including the 6-min walk test (6MWT), Fugl–Meyer assessment of the lower extremity (FML-LE), modified Ashworth scale (MAS), and Berg balance scale (BBS), were performed to evaluate participants’ gait, lower limb function, and balance. The clinical assessments were performed by the same licensed physical therapist. The order of the clinical tests was conducted randomly before training.

### Electrical Impedance Myography Assessment

**FIGURE 1:**
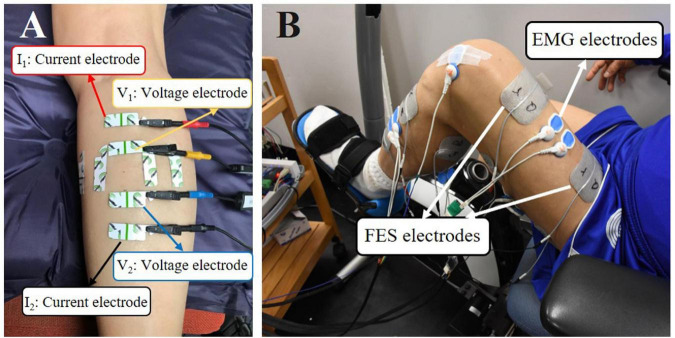
**(A)** Demonstration of EIM assessment on the MG muscle. The outer two electrodes (the red and black ones) were current electrodes, and the inner two electrodes (the yellow and blue ones) were voltage electrodes. The longitudinally and transversely arranged electrodes were used, and only results from longitudinal measurement were used for statistical analysis. **(B)** A male subject was performing FES-assisted cycling training. The FES was triggered by muscle activation signals captured by surface electromyography (sEMG) electrodes.

The composition of the four muscle groups was assessed by EIM (Imp SFB7 Impedimed, Inc., Sydney, NSW, Australia). The muscle groups of paretic side were assessed before and after training, and the muscles of the non-paretic side were assessed before training only. The muscle belly of each muscle group was the target area for the evaluation (Figure 1).

Before each test, an alcohol pad was applied to clean the skin. Four electrodes (an inner pair of voltage electrodes and one pair of current electrodes on the outer regions) were attached linearly along with the muscle. The distance of the inner pair of current electrodes was 30 mm and that of the outer two current electrodes was 80 mm. The dimensions of the electrodes were 2.5 cm × 1 cm (Figure 1A). Each target muscle was measured three times. The acquired values were averaged for statistical analysis.

### Functional Electrical Stimulation-Assisted Cycling

The cycling device consisted of an interactive cycling system with a motor (UIRobot UIM241Co4P-IE/57-76, Shanghai, China) and a pair of pedal meters (Power Tap P1 Pedal Meter, CycleOps, Madison, WI, United States). Every recruited subject participated in a 40-min FES-assisted cycling training session (two 15-min sessions with 10 min of rest). Subjects were asked to abstain from any high-intensity exercise on the day before the experiment. During the training sessions, subjects were seated on the chair of the system and pedaled with FES assistance. A goniometer was applied to measure the angles of both knees while adjusting the distance between the chair and the cycling system to ensure a maximum knee extension angle of approximately 140–150° when pedaling (Hu et al., 2021).

A programmable FES device (P-9632FineCure Easy Walker, Guangzhou, China) with four portable channels, including electrodes (AXLEGAARD PALS electrodes, Axelgaard Manufacturing Company, CA, United States, Neurostimulation Electrodes, United States), was employed to provide muscle stimulation. Four pairs of electrodes were attached to the skin surface over the muscle belly of the four paretic lower limb muscles. For RF, MG, and BF, 5 cm × 8 cm surface electrodes were used, and 5 cm × 5 cm surface electrodes were used for TA due to the smaller area on the skin surface. The FES pulse bandwidth was set between 100 and 420 μs, and the stimulation frequency was 20 Hz. The stimulation intensity could be modulated between 0 and 100 mA. The intensity of the stimulation of these 4 muscles was determined by taking the FES amplitude that induced a visible twitch on the corresponding muscles without causing any pain or discomfort to the subjects (Figure 1B). Detailed information about FES-assisted cycling training can be found in our previous study (Hu et al., 2021).

### Data and Statistical Analysis

The EIM parameters *X*, *R*, and θ were obtained at frequencies of 5–1,000 kHz and exported for analysis. Only EIM parameters collected at a high frequency (100 kHz) were analyzed to avoid interference from the high impedance of the electrode–skin contact surface (Li L. et al., 2017). The clinical assessment scale scores were recorded for correlation analysis.

The parameters *R*, *X*, and θ (mean ± SE) were analyzed and reported. Paired *t*-test analysis was conducted to compare the *R*, *X*, and θ values between the pre- and post-training sessions, as well as between the paretic and non-paretic sides for normally distributed data. The Wilcoxon matched-pairs signed rank sum test was used for non-normally distributed data. The Shapiro–Wilk test was applied to verify the normality of the data. Correlation analysis was conducted between the clinical scale scores (i.e., FMA-LE, BBS, 6MWT, and MAS) and the pre–post training differences in the *R*, *X*, and θ values of the four muscle groups (difference value = post-training value - pre-training value on the paretic side). Pearson’s correlation analysis was employed for normally distributed data, while Spearman’s correlation analysis was employed for non-normally distributed data. Statistical significance was defined as *p* ≤ 0.05 (two-tailed). Statistical analysis was conducted using SPSS 23 software (IBM Inc., WA, United States).

## Results

### Comparison Between the Paretic and Non-paretic Muscles Before Training

**FIGURE 2:**
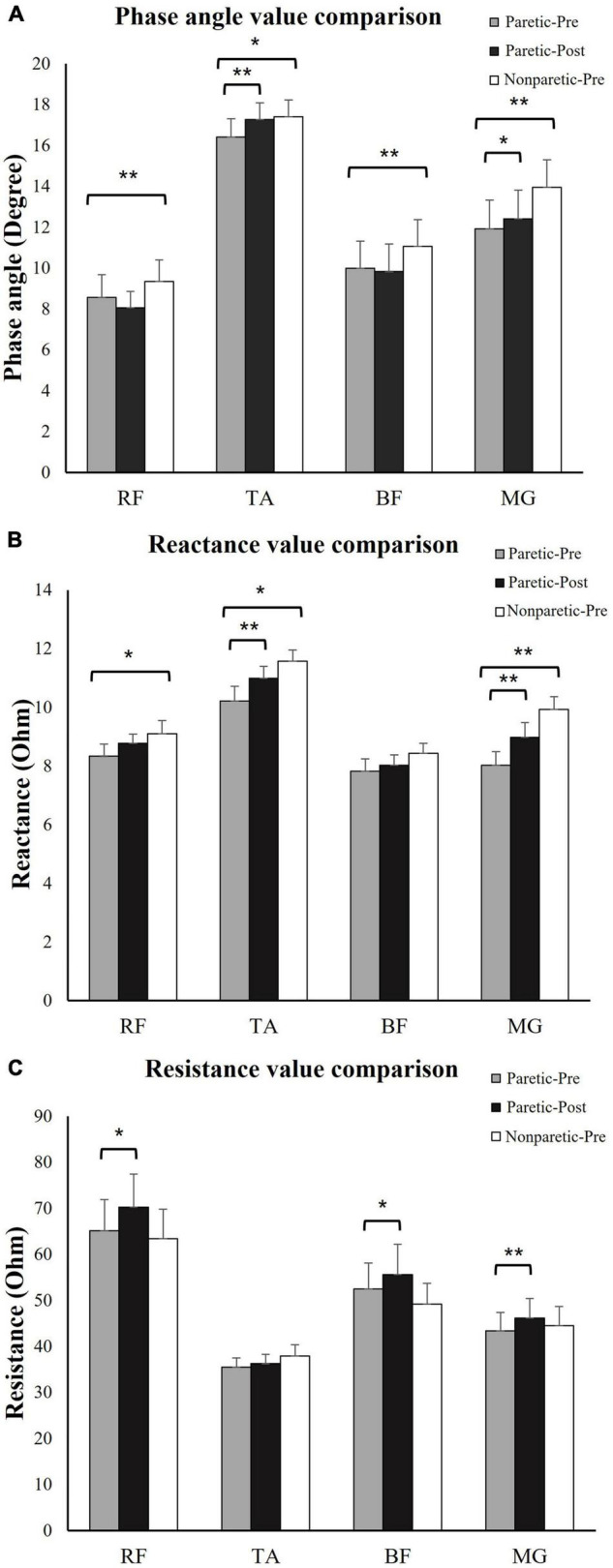
Electrical impedance myography (EIM) parameters comparison. **(A)** Phase angle (θ) comparison: θ was significantly lower in paretic BF, TA, BF, and MG compared to non-paretic side. Immediately after training, θ significantly increased in TA and MG. **(B)** Reactance (*X*) value comparison: *X* was significantly lower in paretic BF, TA, and MG compared to non-paretic side. Immediately after training, *X* significantly increased in TA and MG. **(C)** Resistance (*R*) value comparison: *R* significantly increased in RF, BF, and MG after training. *p ≤ 0.05, **p ≤ 0.01. RF, rectus femoris; TA, tibias anterior; BF, biceps femoris; MG, medial head of gastrocnemius; paretic-pre, paretic muscle pre-training; paretic-post, paretic muscle post-training; non-paretic-pre, non-paretic muscle pre-training.

The *R*, *X*, and θ of the four tested muscle groups were compared between the paretic and non-paretic sides. θ was significantly smaller in all of the measured muscles on the paretic side compared to the non-paretic side (RF, paretic: 8.57 ± 1.1, non-paretic: 9.35 ± 1.0, *p* = 0.004; TA, paretic: 16.42 ± 1.0, non-paretic: 17.40 ± 0.8, *p* = 0.046; BF, paretic: 10.08 ± 1.4, non-paretic: 11.07 ± 1.3, *p* = 0.001; MG, paretic: 11.92 ± 1.4, non-paretic: 13.94 ± 1.4, *p* = 0.003). *X* was significantly lower in the paretic RF (paretic: 8.33 ± 0.4, non-paretic: 9.10 ± 0.4, *p* = 0.019), TA (paretic: 10.21 ± 0.5, non-paretic: 11.58 ± 0.4, *p* = 0.023), and MG (paretic: 8.02 ± 0.5, non-paretic: 9.93 ± 0.4, *p* < 0.001). No significant difference in *R* was present between the paretic and non-paretic muscles (Figure 2).

### Comparison Between the pre- and Post-training Sessions at the Paretic Side

Statistical analysis revealed that immediately after the training session at the paretic muscles, the *R*-values for the RF (pre-training: 65.09 ± 6.8, post-training: 70.21 ± 7.2, *p* = 0.029), BF (pre-training: 53.04 ± 6.4, post-training: 55.60 ± 6.7, *p* = 0.047), and MG (pre-training: 43.37 ± 4.2, post-training: 46.19 ± 4.4, *p* = 0.006) were significantly increased. The *X* values were significantly increased for the TA (pre-training: 10.21 ± 0.5, post-training: 10.99 ± 0.4, *p* = 0.023) and MG (pre-training: 8.02 ± 0.5, post-training: 8.98 ± 0.5, *p* < 0.001). The θ values were significantly increased for the TA (pre-training: 16.42 ± 1.0, post-training: 17.26 ± 1.0, *p* = 0.002) and MG (pre-training: 11.92 ± 1.4, post-training: 12.40 ± 1.4, *p* = 0.021) (Figure 2). No significant change in other impedance variables was found in the RF or BF (Figure 2).

### Analysis of the Correlation Between the Clinical Scale Scores and Impedance Parameters

**FIGURE 3:**
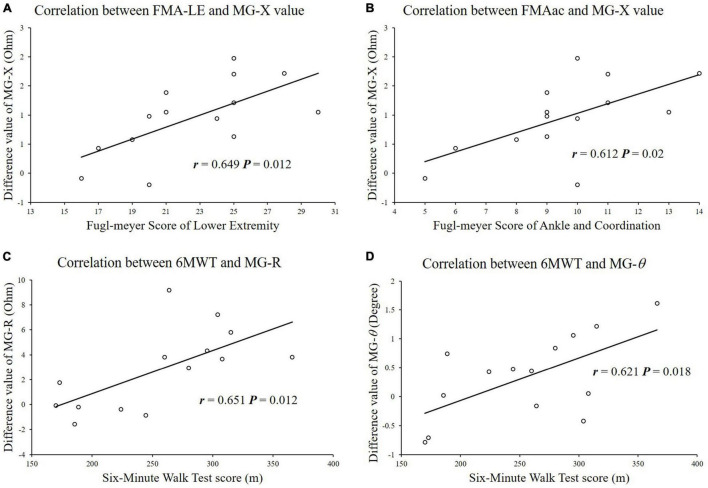
Correlation analysis between impedance parameters and functional clinical scale scores. **(A)** FMA-LE scores were significantly correlated with difference of reactance values in MG. **(B)** FMAac scores were significantly correlated with difference of reactance values in MG. **(C)** 6MWT scores were significantly correlated with difference of resistance values in MG. **(D)** 6MWT scores were significantly correlated with difference of reactance values in MG.

The correlations between the clinical scale scores and the changes in impedance parameters (Δ*R*, Δ*X*, and Δθ) were analyzed (Figure 3). For the MG, the Δ*X* value was significantly correlated with the FMA-LE scores (*r* = 0.649, *p* = 0.012), and the Δ*X* value was significantly correlated with the FMA score of the ankle joint and coordination (*r* = 0.612, *p* = 0.02). The Δ*R* and Δθ values were significantly correlated with the 6MWT scores (X-6MWT: *r* = 0.651, *p* = 0.012, θ-6MWT: *r* = 0.621, *p* = 0.018).

## Discussion

The present study found significant electrical impedance changes in muscles immediately after FES-assisted cycling training. The study also explored the correlation between changes in impedance and clinical scores.

### Immediate Effects of Functional Electrical Stimulation-Assisted Cycling

The present results indicated significant elevation of the *R*, *X*, and θ values of different muscle groups (Figure 2). Hwang investigated the training effects of electrical stimulation combined with exercise on muscle structure changes in chronic stroke survivors. The authors reported a significant increase in muscle pennation angle and muscle thickness (Hwang et al., 2015). Nevertheless, the quantity of myofibers might not be altered immediately after a single training session. The observed changes in the myofiber structure may be due to repetitive muscle contraction. An increase in muscle fiber size would lead to elevations in the *X* and θ values (Rutkove et al., 2002). During voluntary muscle contractions and FES-assisted muscle contractions, temporary hypertrophy and swelling of individual muscle fibers and muscle cross-sectional area (CSA) increased (Konopka and Harber, 2014). The swelling in individual muscle fibers did not fade immediately after a long duration of repeated contractions (Yasuda et al., 2015). The muscle membrane area enlarged within the tissue, which increased the momentary charge storage capability of the tissue and created a greater time shift when an alternating current passed through the muscle (Shiffman and Rutkove, 2013). In addition, muscle fibers were arranged more regularly and compactly during the period of temporary hypertrophy because the swollen muscle fibers occupied more space and squeezed the intramuscular tissue aside. As a result, when performing EIM, the electrical current flowed more through the cytomembrane area of the muscle when passing between the two voltage electrodes than it did before the cycling training. As indicated in previous studies, *X* and θ value alterations are related to muscle CSA and muscle fiber size (Li et al., 2013, 2014; Li X. et al., 2017). Hu and coworkers also applied ultrasound and EIM simultaneously and found that muscle thickness and fascicle length positively correlated with the *R* and θ values (Hu et al., 2019). The observed results suggested that the increased *X* and θ values indicated muscle hypertrophy and CSA increased immediately after FES cycling training. However, the present study explored only the immediate effects of FES-assisted training and did not assess the cumulative training effects of prolonged muscle contraction. In future EIM studies, multiple sessions of training need to be conducted to assess whether FES-assisted cycling training contributes to muscle CSA and myofiber increases as well as functional improvement.

Muscle fatigue after a long duration of training might also play a role in muscle property alterations. Muscle fatigue is a complicated psychophysiological state that involves muscle weakness, pain, and a reduction in force production (Romani, 2008). It was reported that persistent force generation and repeated contraction influence the intramuscular environment and may also be associated with hypertrophy by causing hypoxic conditions and the accumulation of metabolic subproducts (Takarada et al., 2000). Li revealed that due to a significant accumulation of metabolites and intracellular fluids during muscle contraction, the conductivity improved and *R* decreased (Li L. et al., 2016). Todd applied EIM to biceps tissue and demonstrated a significant decrease in *R* and *X* values during a fatiguing exercise protocol (Freeborn and Fu, 2019). The alteration of impedance parameters observed in the present study might be related to the accumulation of metabolites and intracellular fluids associated with muscle fatigue. However, the impedance parameters in the present study increased immediately after the training, which contradicts the findings reported in the study by Li and Todd. The reason might be the difference in the study setup since muscle contractions during cycling induced many more muscle length changes than the isometric contractions in Li’s study. The continuous dynamic muscle contractions generated by FES in the present study influenced the internal muscle tissues substantially, and the insulating boundaries (connective tissues) were relatively shifted and twisted (Shiffman et al., 2003). Repetitive muscle contractions led to more significant morphological alterations than the accumulation of metabolites, which might be the reason for the increase in *R* and *X* values. Further investigations of metabolism with a combination of different measuring techniques might be required to substantiate the findings, as multiple types of pathological changes could contribute to the results. Nonetheless, this is an inference based on the results of the present study and requires further investigation.

### Clinical Relevance

The alteration of muscle impedance properties after training was positively correlated with clinical scores, which suggested that residual motor function might play a role in muscle intrinsic property alterations after FES-assisted cycling training. Stroke survivors with higher motor function might perform cycling with more voluntary muscle contraction than those with lower motor function levels (Figure 3). Detection of the immediate training effects using EIM also suggested the feasibility of evaluating the cumulative training effect delivered by FES-assisted cycling training. Therefore, in our future study, we would aim to design a study to clarify the effects of long-term cumulative training and explore the underlying mechanism of the intrinsic muscle property alterations induced by physical training.

### Limitations

There are a number of limitations in the present study. First, the sample size of this study was limited, which might result in bias. Second, in the present study, only the muscle alterations immediately after a single session of training were examined, and we could not clarify the contribution of voluntary exercise vs. FES to the changes based on the observed results. It would be interesting to explore the effect of voluntary contraction and FES, which would provide more guidance for clinical treatment. Thus, additional randomized controlled studies with large sample sizes and multiple training sessions are required to elucidate the effect of FES and cycling training. In addition, there were differences in the parameter changes for different muscle groups. Thus, to explore muscle performance changes, in future studies, we will record the stimulation intensity for each muscle group and conduct additional quantitative assessments of muscle function, such as by using handheld dynamometers to measure muscle strength, ultrasound to measure muscle architecture, and electromyography techniques to measure muscle activation.

## Conclusion

This brief report illustrated the immediate effect of FES-assisted cycling training on muscle intrinsic properties, as well as the correlation between clinical scores and intrinsic muscle property alterations in chronic stroke survivors. There were measurable changes in muscle impedance in four muscle groups of interest after chronic stroke, and these changes were related to motor function. The results showed that EIM is a sensitive technique that can be used to detect intrinsic muscle property alterations immediately after 40 min of FES-assisted cycling training. Therefore, EIM could provide a novel understanding of the mechanism of muscle function improvements from the perspective of intrinsic muscle properties after FES and cycling training. This could assist clinicians in diagnosing and evaluating muscle changes after FES training in chronic stroke survivors and provide alternative options for therapists providing rehabilitation therapy.

## Data Availability Statement

The datasets analyzed during the current study are available from the corresponding author upon reasonable request.

## Ethics Statement

The studies involving human participants were reviewed and approved by the Joint Chinese University of Hong Kong-New Territories East Cluster (CUHK-NTEC) Clinical Research Ethics Committee (Ref. no: 2016.093-T). The patients/participants provided their written informed consent to participate in this study.

## Author Contributions

LL and RT conceived and designed the study, made contributions to experiments, and reviewed and edited the manuscript. CH and KL performed the experiments. CH and LL wrote the manuscript. All authors read and approved the final manuscript.

## Conflict of Interest

The authors declare that the research was conducted in the absence of any commercial or financial relationships that could be construed as a potential conflict of interest.

## Publisher’s Note

All claims expressed in this article are solely those of the authors and do not necessarily represent those of their affiliated organizations, or those of the publisher, the editors and the reviewers. Any product that may be evaluated in this article, or claim that may be made by its manufacturer, is not guaranteed or endorsed by the publisher.
